# Freeze–Thaw-Induced Deterioration and Failure Mechanisms of Permeable Concrete in Cold Regions

**DOI:** 10.3390/ma19183880

**Published:** 2026-09-11

**Authors:** Zirui Guo, Zhongzhi Guan, Yongzhen Zhang, Ting Li, Riguang Chi, Yong Sun, Zhiqiang Chen

**Affiliations:** 1 School of Energy and Architecture Engineering, Harbin University of Commerce, Harbin 150023, China; ziruiguo@126.com (Z.G.); yongzhenzhang0613@163.com (Y.Z.); 103367@hrbcu.edu.cn (T.L.); chiriguang@163.com (R.C.); 2 School of Civil Engineering, Heilongjiang University of Science and Technology, Harbin 150022, China; 3 State Key Laboratory of Urban Water Resources and Water Environment, Harbin Institute of Technology, Harbin 150090, China; 102920@hrbcu.edu.cn

**Keywords:** permeable concrete; freeze–thaw cycles, porosity; physical and mechanical properties, computational fluid dynamics (CFDs)

## Abstract

**Highlights:**

Freeze–thaw degradation of permeable concrete was quantified up to 120 cycles. Damage evolved from paste peeling and microcracks to pore-structure failure. BPN remained above 70 after freeze–thaw cycles, indicating high skid resistance. Higher porosity accelerated strength loss, permeability decay, and pore expansion. Numerical sensitivity analysis explored the influence of porosity on thermal response and phase-change duration under idealized conditions.

**What are the main findings?**

- Freeze–thaw damage of permeable concrete develops progressively: starting from cement paste peeling and microcrack propagation, then evolving into pore structure degradation and local skeleton failure; higher initial porosity accelerates mass loss, porosity growth, compressive strength, and permeability attenuation after up to 120 freeze–thaw cycles.
- After 120 freeze–thaw cycles, all specimens have mass loss below 1%, compressive strength drops 5.5–12.9%; all groups retain BPN above 70 and maintain decent permeability, meeting basic pavement anti-slip and drainage requirements for cold-region roads.
- CFD simulations prove permeable concrete shows three-stage temperature responses in both freezing and thawing; rising porosity reduces effective thermal conductivity, extends phase-change duration, amplifies internal temperature gradients, and aggravates thermo–mechanical coupling frost damage.

**What are the implications of the main findings?**

- Optimizing the pore structure (appropriately lowering design porosity) is the core measure to improve the long-term frost resistance and service durability of cold-region pervious pavement.
- Even after long-term freeze–thaw cycling, permeable concrete still satisfies standard anti-slip and drainage performance, verifying its basic applicability for cold-area sponge city walkways and light traffic pavements.
- Numerical sensitivity analysis can provide qualitative insights into the thermal response of porous pavement materials, but quantitative prediction requires experimental temperature validation.

**Abstract:**

To investigate the performance degradation patterns and underlying damage mechanisms of permeable concrete under freeze–thaw cycles in cold regions, permeable concrete with varying porosities was selected as the research subject. A total of 120 rapid low-temperature freeze–thaw cycles were conducted. The evolution of porosity, mass loss, skid resistance, permeability, and compressive strength was systematically analyzed. Exploratory numerical simulations, conducted under idealized assumptions, suggest that rising porosity may reduce effective thermal conductivity, extend phase-change duration, and amplify internal temperature gradients—trends that are consistent with the observed porosity-dependent frost damage but require experimental temperature validation for quantitative confirmation. With the increase in freeze–thaw cycles, mass loss and porosity continuously increase, while compressive strength and permeability gradually decrease. After 120 cycles, the mass loss of all specimen groups was below 1%, with compressive strength decreasing by 5.5% to 12.9%. Despite this, the specimens maintained good permeability and skid resistance. Numerical simulations indicate that permeable concrete exhibits a three-stage temperature response during both freezing and thawing processes. An increase in porosity reduces the material’s effective thermal conductivity, prolongs the phase transition duration, and intensifies the internal temperature gradient, thereby amplifying the thermo–mechanical coupling damage effects. Therefore, optimizing the pore structure is crucial for improving the long-term service performance of permeable pavements in cold regions.

## 1. Introduction

Permeable pavement serves as the core material for achieving the functions of “infiltration, detention, retention, and utilization” in sponge city design. Its highly interconnected porous structure effectively facilitates rainwater infiltration, reduces surface runoff, and mitigates the urban heat island effect. However, as the development of sponge cities progresses into colder regions, the frequent occurrence of freeze–thaw cycles during winter has become a critical bottleneck that restricts their long-term performance [1,2]. The permeability, mechanical strength, and durability of permeable concrete are highly dependent on its internal pore structure. Under alternating freeze–thaw conditions, the internal pressure generated by the expansion of pore water freezing can easily lead to surface spalling, deterioration of mechanical properties, and a decline in permeability, which pose a significant threat to the stability and lifespan of the pavement structural integrity [3,4,5,6]. Therefore, understanding the performance evolution of permeable concrete in cold regions under freeze–thaw cycles holds significant engineering importance.

Current research on permeable concrete primarily focuses on material composition, mechanical properties, permeability characteristics, and durability [7]. Guan et al. [8] identified that prolonged clogging, insufficient strength, and inadequate environmental adaptability are the main bottlenecks limiting the application of permeable pavements in sponge cities. In terms of optimizing microstructure and mix design, Shan et al. [9] analyzed the intrinsic relationship between the pore characteristics of permeable concrete and its permeability performance, clarifying the controlling role of pore connectivity and pore size distribution on permeability stability. Mahalingam et al. [10] established the relationship between aggregate gradation and the amount of binding materials concerning the strength and porosity of permeable concrete. Regarding durability in complex environments, Zaetang and Hua et al. [11,12] utilized recycled aggregates to prepare permeable concrete, verifying the feasibility of using recycled aggregates in permeable pavements and highlighting the significant impact of recycled aggregate quality on mechanical properties and resistance to sulfate corrosion. Song et al. [13] employed ultrasonic methods for non-destructively predicting the strength degradation of permeable concrete under sulfate wet–dry cycles, providing a methodology for assessing performance in corrosive environments. Yan et al. [14] investigated the mechanical properties and lifespan predictions of composite modified permeable concrete after seawater erosion, offering support for applications in harsh coastal environments. Zheng et al. [15] explored the degradation mechanisms of thermal, physical, and mechanical properties of permeable concrete under freeze–thaw cycles, noting that micro-crack propagation and interfacial damage are central to performance degradation.

In summary, current research primarily focuses on ambient temperature or chemically corrosive environments. Although some studies address freeze–thaw cycles, they mainly focus on ordinary concrete or rock, and there is a notable lack of systematic exploration regarding the performance degradation patterns, damage evolution mechanisms, and the temporal and spatial distribution of internal temperature fields in permeable concrete with varying designed porosities under freeze–thaw conditions [16,17,18,19,20]. In cold regions, the interconnected pores in permeable concrete are prone to saturation, and the phase change expansion and frost heave stresses caused by repeated freeze–thaw cycles can lead to interface delamination, crack propagation, and even structural failure [21,22]. Among these, porosity serves as a critical parameter governing freeze–thaw damage, directly influencing the material’s saturation degree, heat transfer rate, and temperature gradient, which results in significant differences in temperature responses and damage modes among specimens with varying porosities [23,24,25,26]. Given that traditional experiments struggle to capture the evolution of internal temperature fields and heat transfer paths in real-time, the introduction of CFD numerical simulation can effectively overcome this limitation, enabling visualizations of temperature fields and quantitative analyses of hydrothermal coupling behavior during freeze–thaw processes [27,28,29,30]. Wen et al. [31] investigated fiber-reinforced permeable concrete, calibrating experimental permeability coefficients with numerical simulation results, thereby clarifying the mechanisms by which fiber modification regulates concrete pore structure and optimizes permeability performance. Yang et al. [32] conducted durability performance experiments and numerical simulations on waste glass sand concrete subjected to alternating dry–wet and freeze–thaw conditions, effectively revealing the accelerated damage mechanisms of concrete structures due to multi-field coupling. Gao et al. [33] focused on coal gangue-based concrete in cold regions, using numerical simulations to analyze the internal pore expansion, damage accumulation, and strength degradation characteristics at various freeze–thaw cycle counts, thereby revealing the freeze–thaw degradation mechanisms of materials in cold environments. Wang et al. [34] conducted a numerical simulation study on freeze–thaw damage at the mesoscopic scale of hydraulic concrete, quantifying the relationships between the number of freeze–thaw cycles and the distributions of mesoscopic stress, pore structure deformation, and internal damage evolution.

In summary, this paper focuses on permeable concrete with varying porosities. Through accelerated freeze–thaw cycle experiments, it systematically examines the impact of the number of freeze–thaw cycles and porosity on the physical and mechanical properties of permeable concrete, elucidating the deterioration mechanisms and failure modes of permeable concrete under freeze–thaw conditions. Additionally, by utilizing CFD numerical simulation methods, a heat transfer model for the freeze–thaw process of permeable concrete is developed. This model simulates the temperature field distribution of specimens with differing porosities during the freezing and melting phases, as well as the variations in maximum and minimum temperatures over time and the associated heat transfer characteristics. It reveals the evolutionary mechanisms of temperature field dynamics and the thermohydraulic coupling effects on damage development under freeze–thaw cycling conditions.

## 2. Experimental Design and Methodology

In this work, 120 low-temperature accelerated freeze–thaw cycle tests were carried out on self-prepared permeable concrete with varied porosities to systematically evaluate its skid resistance, permeability coefficient, and compressive strength. While these laboratory cycles roughly correspond to one-to-two-year cumulative effective freeze–thaw events for cold-region pavements, they fail to reproduce the complex coupling of dynamic-traffic-induced fatigue, de-icing-salt-triggered chemical corrosion, and time-dependent moisture-content variation under practical service; thus, experimental results cannot be used.

### 2.1. Experimental Materials

The primary raw materials utilized in this experiment consist of cementitious materials, aggregates, and additives. The cementitious materials include P·O 52.5 ordinary Portland cement produced by Yatai Group Harbin Building Materials Co., Ltd. (Harbin, China), supplemented with fly ash from Harbin Qida Building Materials Co., Ltd. (Harbin, China). The fly ash has a specific surface area of 455 m^2^/kg and a loss on ignition of 2.3%, with its chemical composition detailed in Table 1.

Coarse aggregates consist of natural crushed stone produced by Yatai Group Harbin Building Materials Co., Ltd., with a particle size range of 5 to 10 mm and a clay content maintained at 0.35 wt%. The physical performance parameters can be found in Table 2.

The aggregates are ordinary river sand provided by Xinfah Building Materials, with a designed sand ratio of 5%, and the physical performance parameters are presented in Table 3.

A polycarboxylate superplasticizer is used, with a dosage set at 0.30% of the total mass of the cementitious materials, achieving a water reduction rate of at least 25%. This superplasticizer does not contain air-entraining or retarding components, ensuring that the mixture maintains excellent workability under low water-to-cement ratios. This helps to prevent issues such as abnormal pore enlargement or blockage of voids during the formation of permeable concrete.

### 2.2. Mix Ratio Design and Preparation

To meet the practical performance requirements of pervious pavements in cold regions, this study focuses on pervious concrete used for road surfaces. A total of three target porosities (15%, 20%, and 25%) are designed for the experiment. These target porosities were achieved by adjusting the aggregate-to-binder ratio while keeping the single-sized coarse aggregate grading (5–10 mm) and water-to-binder ratio (0.30) constant. The specimens are sized at 200 mm × 100 mm × 60 mm, resulting in a total of 72 specimens prepared. The mix design for the pervious concrete follows the volumetric method outlined in the CJJ/T 135-2009, as shown in Table 4. The measured porosities, determined via the hydrostatic weighing method (detailed in Section 2.3.2), closely matched the target values, with deviations within ±1.2%, ensuring the reliability of the designed porosity levels.

The required quantities of cement (***m_c_***), aggregates (***m_g_***), water (***m_w_***), and additives (***m_a_***) per unit volume are determined by Equation (1):

(1) $$\frac{m_{c}}{\rho_{c}}+\frac{m_{g}}{\rho_{g}}+\frac{m_{w}}{\rho_{w}}+\frac{m_{a}}{\rho_{a}}+p=1$$

where ***ρ_c_***, ***ρ_g_***, ***ρ_w_***, and ***ρ_a_*** represent the apparent densities of cement, aggregate, water, and additives, respectively, while ***P*** denotes the design porosity.

To ensure that the binding materials uniformly envelop the aggregates and prevent the slurry from settling, a two-stage mixing method was employed in the preparation of the permeable concrete for this experiment. Initially, the weighed coarse and fine aggregates were combined with 50% of the mixing water in a forced-action mixer and pre-mixed for 1 min to thoroughly wet the surface of the aggregates. Subsequently, the entire amount of binding materials and water-reducing agent was added, followed by an additional 1 min of mixing, allowing the slurry to initially adhere evenly to the aggregate surfaces and form a slurry shell. Finally, the remaining 50% of mixing water was added, and mixing continued for another 2 min until the mixture attained a uniform color and exhibited a certain degree of cohesiveness, with the total mixing time limited to 4 min. Upon completion of the mixing, the mixture was quickly transferred into standard molds. To ensure that the designed porosity is not compromised, a combination of manual tamping and micro-vibration was used for the compaction process. After demolding at room temperature, the specimens were moved to a standard curing room (at a temperature of 20 ± 2 °C and a relative humidity of ≥95%) for 28 days, in preparation for subsequent performance tests such as freeze–thaw cycling.

### 2.3. Test Methods

#### 2.3.1. Low-Temperature Freeze–Thaw Test

**Figure 1:**
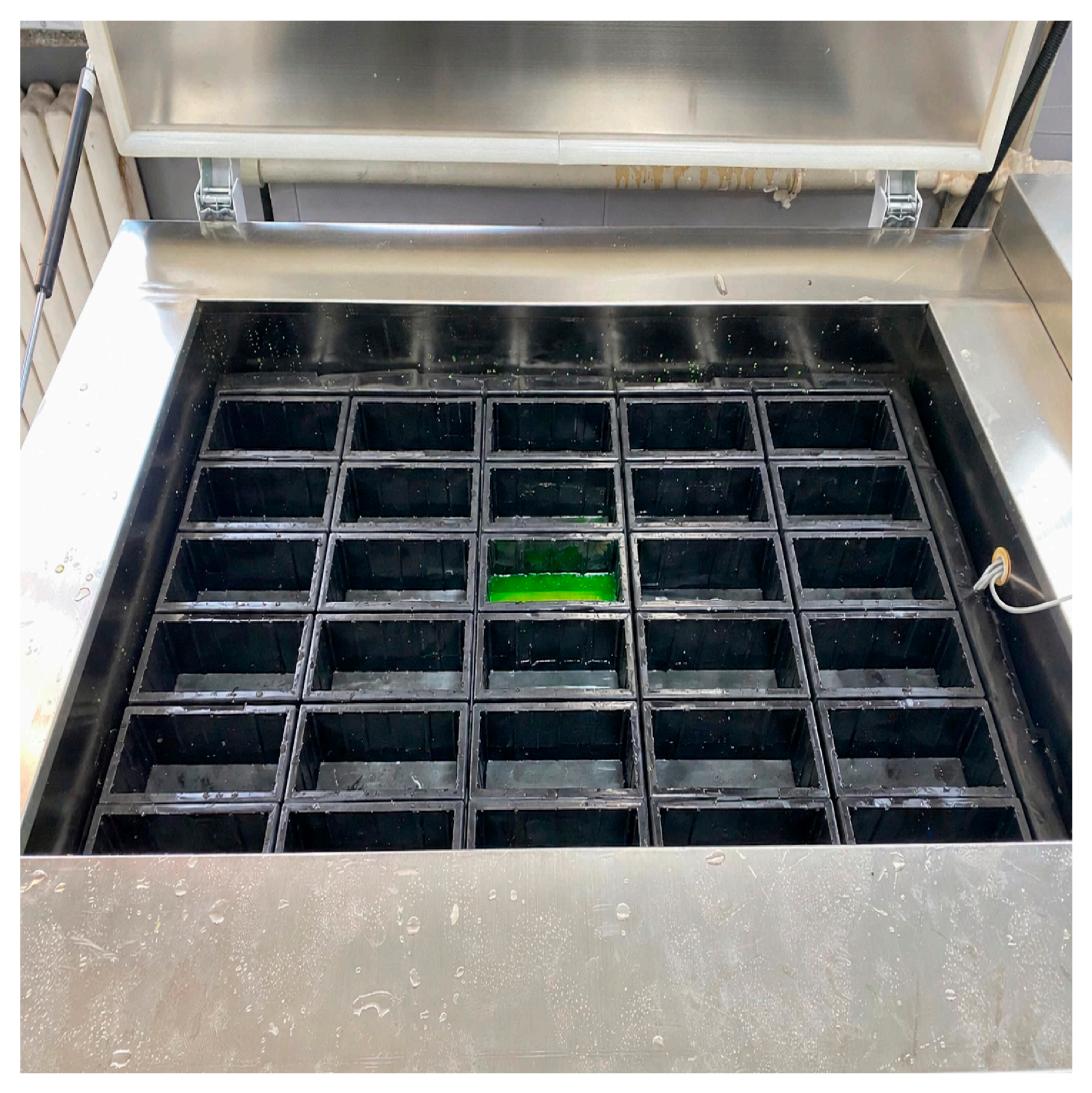
Low-temperature freeze–thaw test.

According to the GB/T 50082-2009 standard, the surface of the permeable concrete and the dust and impurities within its internal pores were brushed off prior to testing. Taking into account the climatic characteristics of cold regions, the temperature range was set between −25 °C (±2 °C) and 5 °C (±2 °C), with freeze–thaw cycling intervals of 30, 60, 90, and 120 cycles [35]. Each freeze–thaw cycle lasted a total of 6 h, with timing initiated once the core temperature within the chamber reached −25 °C, followed by a constant freezing period of 4 h. After the freezing phase concluded, the melting stage commenced, lasting for 2 h, as illustrated in Figure 1.

#### 2.3.2. Porosity Test

**Figure 2:**
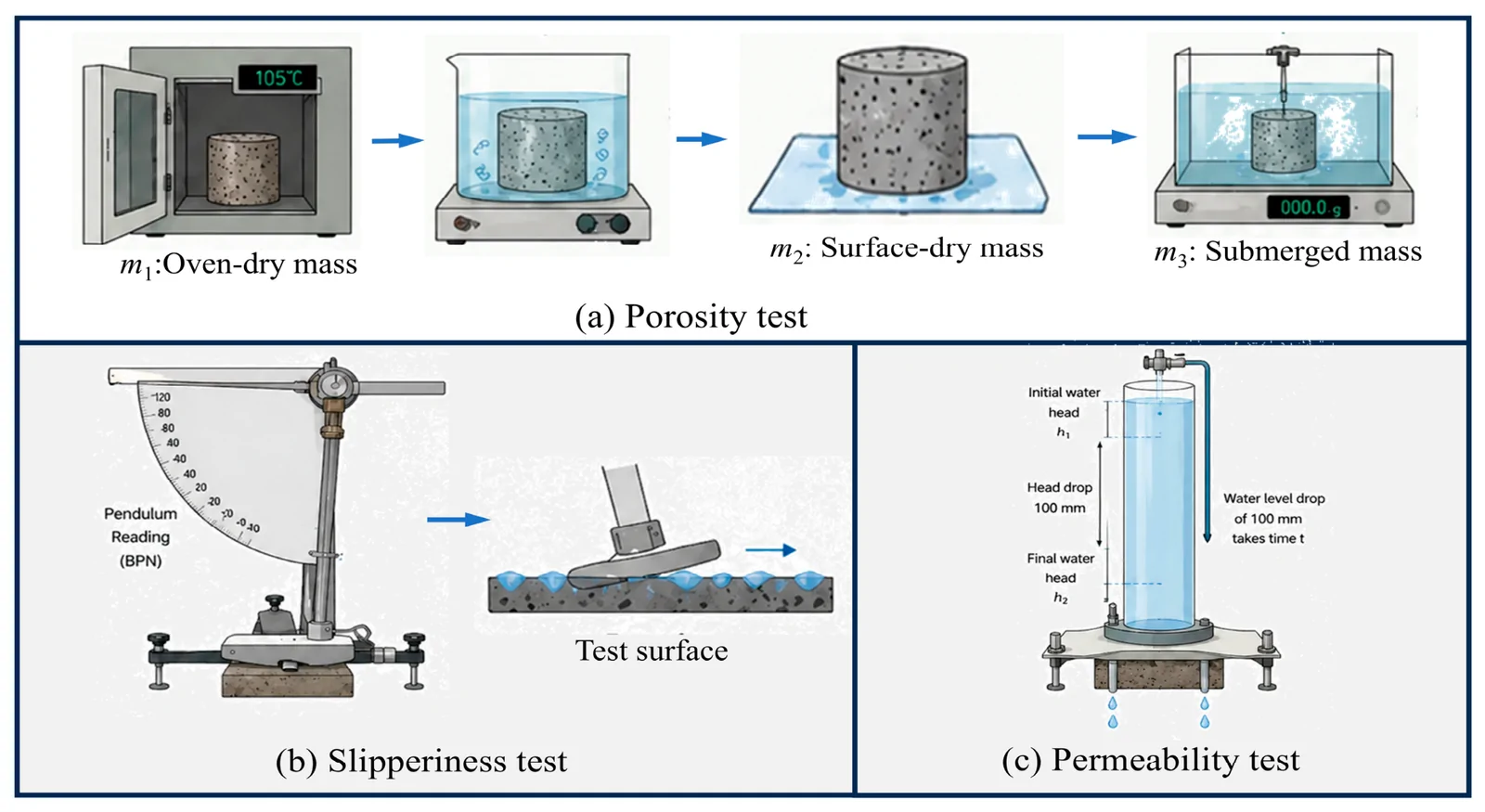
Physical property test.

The porosity determines the number, distribution, and connectivity of the internal pores in permeable concrete, which significantly affects its mechanical properties and permeability on a macroscopic level [36,37]. The porosity of each type of permeable brick is taken as the mean value of three test specimens. The relationships between porosity and water permeability, compressive strength, as well as frost resistance are analyzed and evaluated. To evaluate the inherent relationship between porosity, permeability coefficient, compressive strength, and frost resistance, the effective porosity of the specimens was determined based on Archimedes’ principle, as illustrated in Figure 2a [38]. First, the specimens were placed in an electric hot-air drying oven and dried at 105 °C until they reached a constant weight, then transferred to a desiccator to cool to room temperature. The dry mass at this stage was recorded as ***m***_**1**_. Next, the specimens were immersed in a constant-temperature water bath and boiled for 60 min to ensure complete saturation of the internal pores with water. The specimens were then removed, and the surface free water was rapidly wiped off to achieve a saturated surface-dry condition, with the air mass at this stage recorded as ***m***_**2**_. Finally, the mass of the specimens in water was measured using a hydrostatic balance, recorded as ***m***_**3**_. The porosity of the samples was calculated using Equation (2):

(2) $$p=\frac{\left(m_{2}-m_{1}\right)}{\left(m_{2}-m_{3}\right)}\times 100\%$$

where *P* represents the porosity of the sample (%), ***m***_**1**_ is the mass of the dried sample (g), ***m***_**2**_ is the mass of the saturated sample in air (g), and ***m***_**3**_ is the mass of the saturated sample in water (g).

#### 2.3.3. Slip Resistance Test

The slip resistance of the permeable concrete specimens’ surfaces was tested using a sliding friction tester, as shown in Figure 2b. The testing was conducted on clean and continuously wet surfaces of the specimens. To eliminate errors from the initial contact state, the first sliding reading was discarded. Following this, five valid readings were consecutively recorded at the same position, with a requirement that their range does not exceed 3 BPN; otherwise, a retest was performed. The slip resistance value for each individual specimen was calculated as the arithmetic mean of these five readings. The final slip resistance for each group was taken as the average of the slip values of all specimens, with results rounded to the nearest 1 BPN.

#### 2.3.4. Permeability Test

The permeability of permeable concrete was evaluated using the variable head method, as illustrated in Figure 2c. The porosity of each type of permeable brick was taken as the mean value of three test specimens. Prior to testing, the specimens were fully immersed in water at a temperature ranging from 10 °C to 30 °C for 24 ± 0.25 h, after which they were removed and treated to achieve a saturated surface-dry condition. The sides of the specimens were sealed to ensure that water flow occurred in a one-dimensional vertical direction during the test. Water at a temperature of 10 °C to 30 °C was injected into the testing apparatus, and the time required for the water head to drop by 100 mm was recorded. The permeability coefficient was calculated according to Equation (3):

(3) $$p=\frac{h}{t}$$

where ***P*** represents the permeability (mm/s), ***h*** is the change in water surface position (mm), and ***t*** is the testing time (s).

#### 2.3.5. Compressive Strength Test

**Figure 3:**
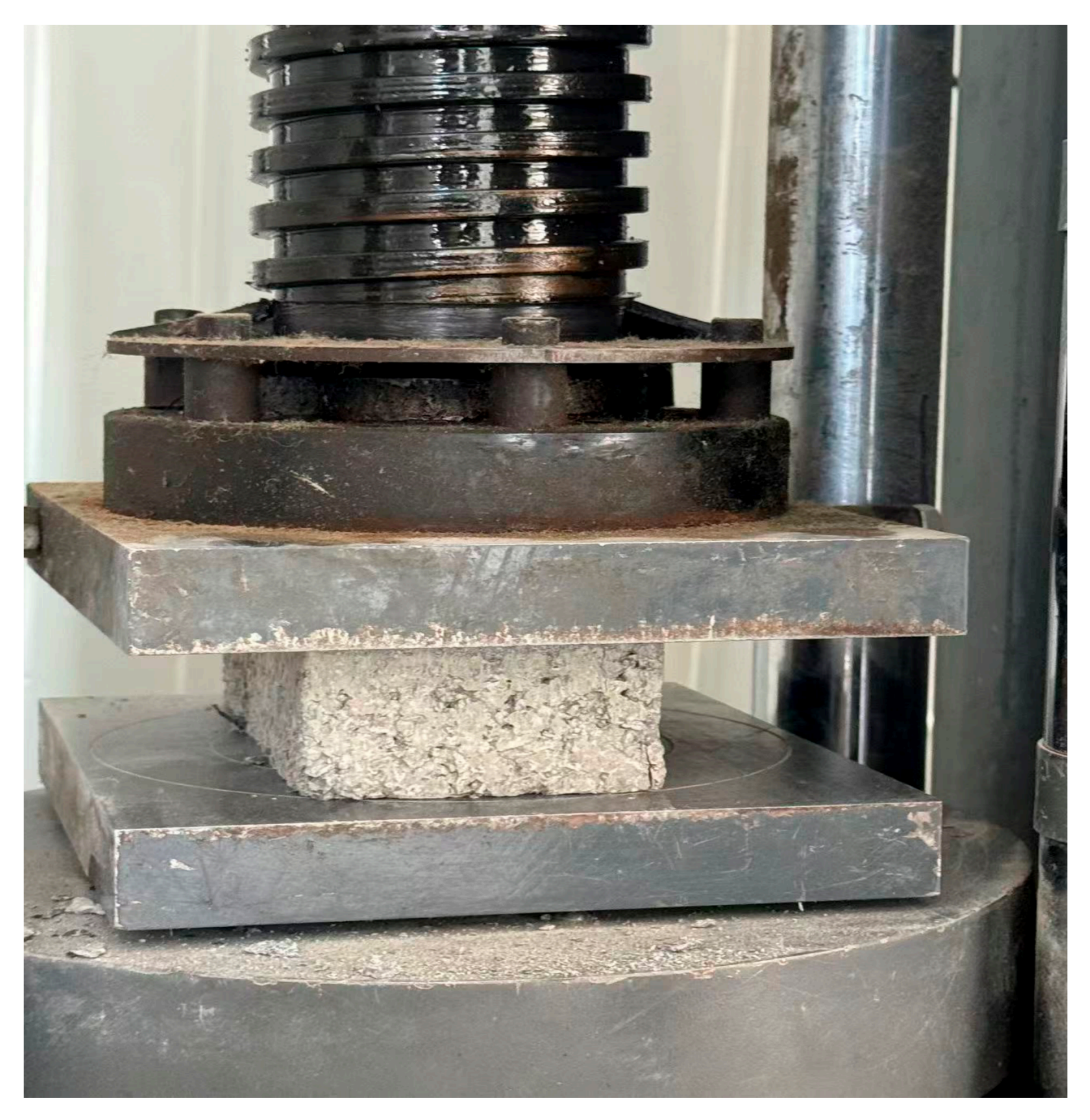
Compression test.

The compressive strength of the specimens was tested using a concrete pressure testing machine, with the formed face serving as the bearing surface, as shown in Figure 3. During the test, a continuous and uniform loading was maintained, with a loading rate set at 0.5 MPa/s. The peak load F at which the specimen failed was recorded. Three specimens were tested for each type of permeable brick, and the test results were averaged.

#### 2.3.6. Microscopic Test

Scanning electron microscopy (SEM) was employed to qualitatively characterize micro-damage morphologies, including paste-aggregate interfacial deterioration, microcrack development, and degradation of hydration products of specimens with different porosities after freeze–thaw cycles, rather than to conduct quantitative image analysis. To eliminate sampling bias caused by accidental local defects, small fragments were excised from the internal pore-wall matrix of specimens, and surface spalling areas were avoided. For each porosity group under each freeze–thaw cycle, multiple observation regions from no less than three replicate specimens were imaged. Only universally observed representative micro-damage features were adopted for subsequent discussion. Prior to testing, the bottom of the samples was polished to a flat surface, and ion sputter gold coating was applied to improve surface electrical conductivity.

## 3. Results and Discussion

### 3.1. Surface Damage Morphology

**Figure 4:**
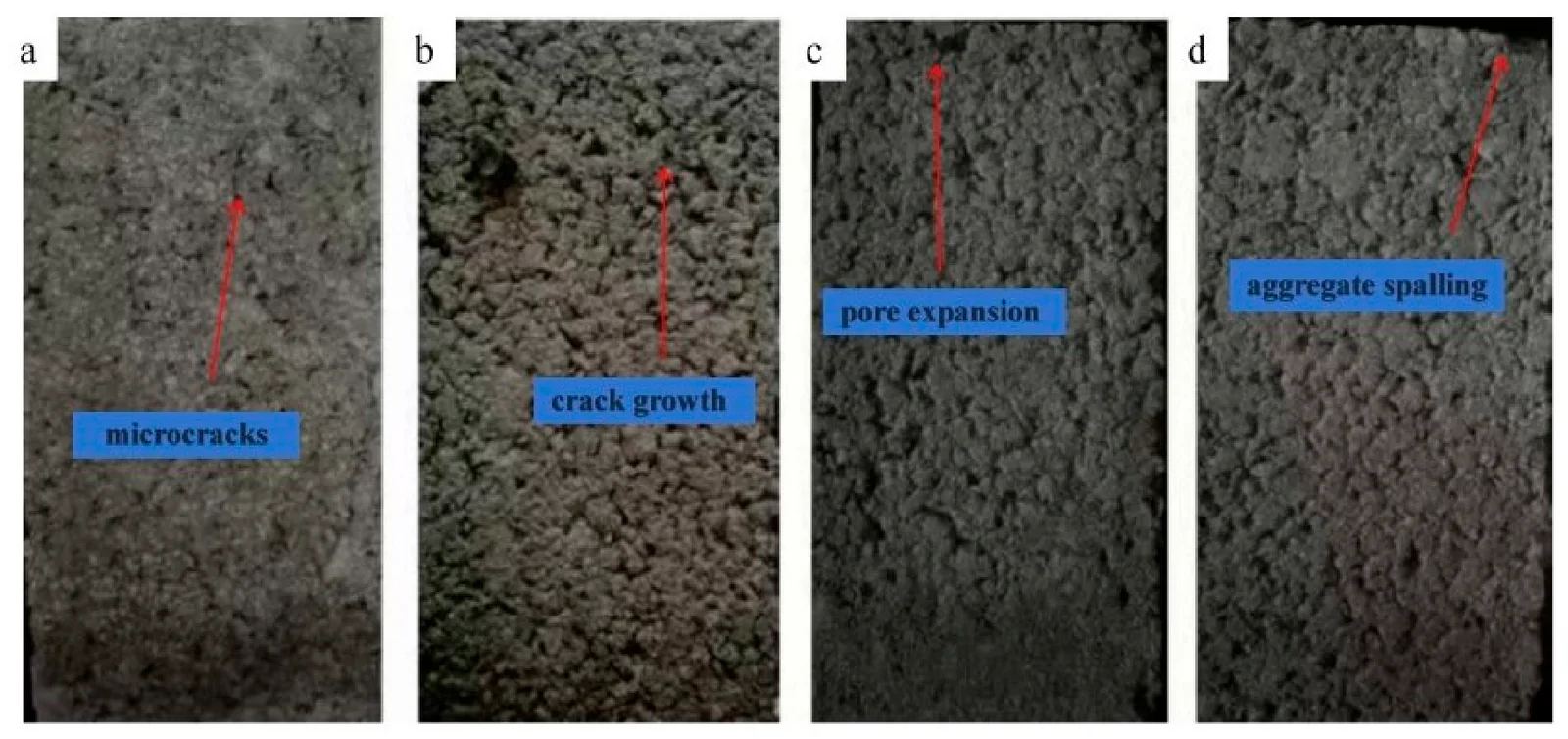
Surface damage morphology: (**a**) microcracks; (**b**) crack growth; (**c**) pore expansion; (**d**) aggregate spalling.

Figure 4 illustrates the evolution characteristics of surface damage in permeable concrete subjected to freeze–thaw cycles. In the early stages of the freeze–thaw cycles, the repeated phase transitions of water within the pores generate freeze–thaw stress, causing microcracks to develop in the internal framework of the specimen. As the number of freeze–thaw cycles increases, these microcracks continue to propagate and intersect, leading to further expansion of existing pores and the onset of delamination of the cementitious material surrounding the aggregate surfaces. By the time the freeze–thaw cycles reach 120, the interfacial bonding strength significantly weakens, ultimately resulting in localized spalling of the coarse aggregates at the surface. This pattern of morphological evolution indicates that the freeze–thaw damage in permeable concrete is fundamentally not due to surface wear, but rather the accumulation of fatigue damage in the internal skeletal structure, which leads to continuous generation, propagation, and interconnection of microcracks, ultimately causing a deterioration of the macroscopic material properties [39,40].

The surface-damage observations imply that higher porosity aggravates freeze–thaw-induced skeleton deterioration. When pursuing high drainage capacity, excessive porosity should be avoided to mitigate skeleton damage, which is one critical prerequisite for achieving a balanced pore-structure design.

**Figure 5:**
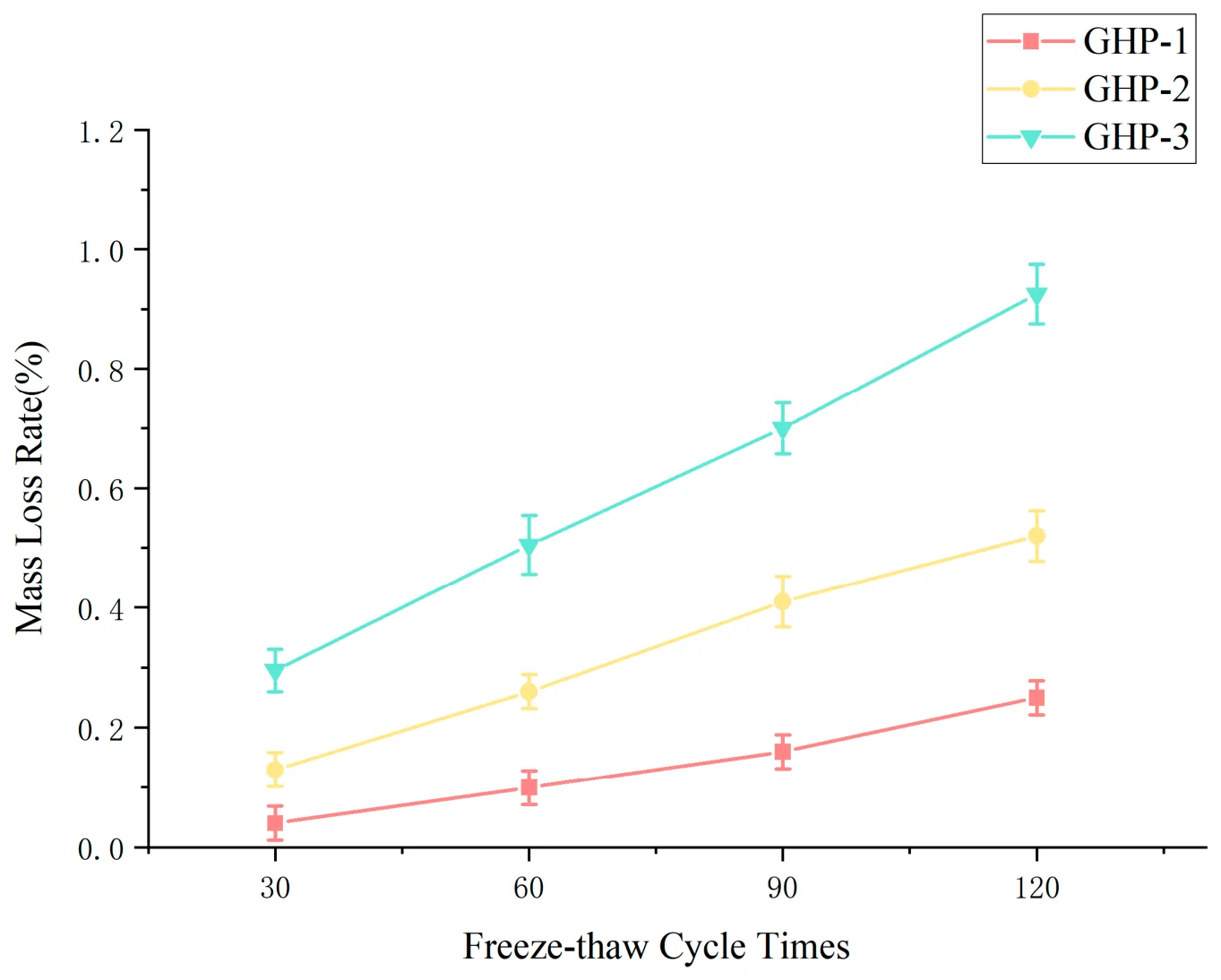
Mass loss evolution.

The mass loss rate of permeable concrete with varying porosities shows a monotonically increasing trend with the number of freeze–thaw cycles, and higher porosity correlates with a faster rate of mass loss, as illustrated in Figure 5. After 30 freeze–thaw cycles, the mass loss rates for the different groups were only 0.10%, 0.15%, and 0.30%, respectively. By the time of 120 cycles, these rates peaked at 0.25%, 0.55%, and 0.95%, respectively. Overall, the mass loss rates for all three groups of specimens after 120 freeze–thaw cycles remained well below the 5% standard limit. This outcome is attributed to the larger internal water-holding capacity and relatively weaker framework of the high-porosity specimens, which exacerbated the growth of internal microcracks due to sustained phase transition expansion forces, while only localized particle or cementitious material shedding occurred on the surface. Consequently, although porosity significantly amplified the adverse effects of internal freeze–thaw damage, the overall esthetic integrity of the material remained relatively intact.

### 3.2. Porosity Evolution

**Figure 6:**
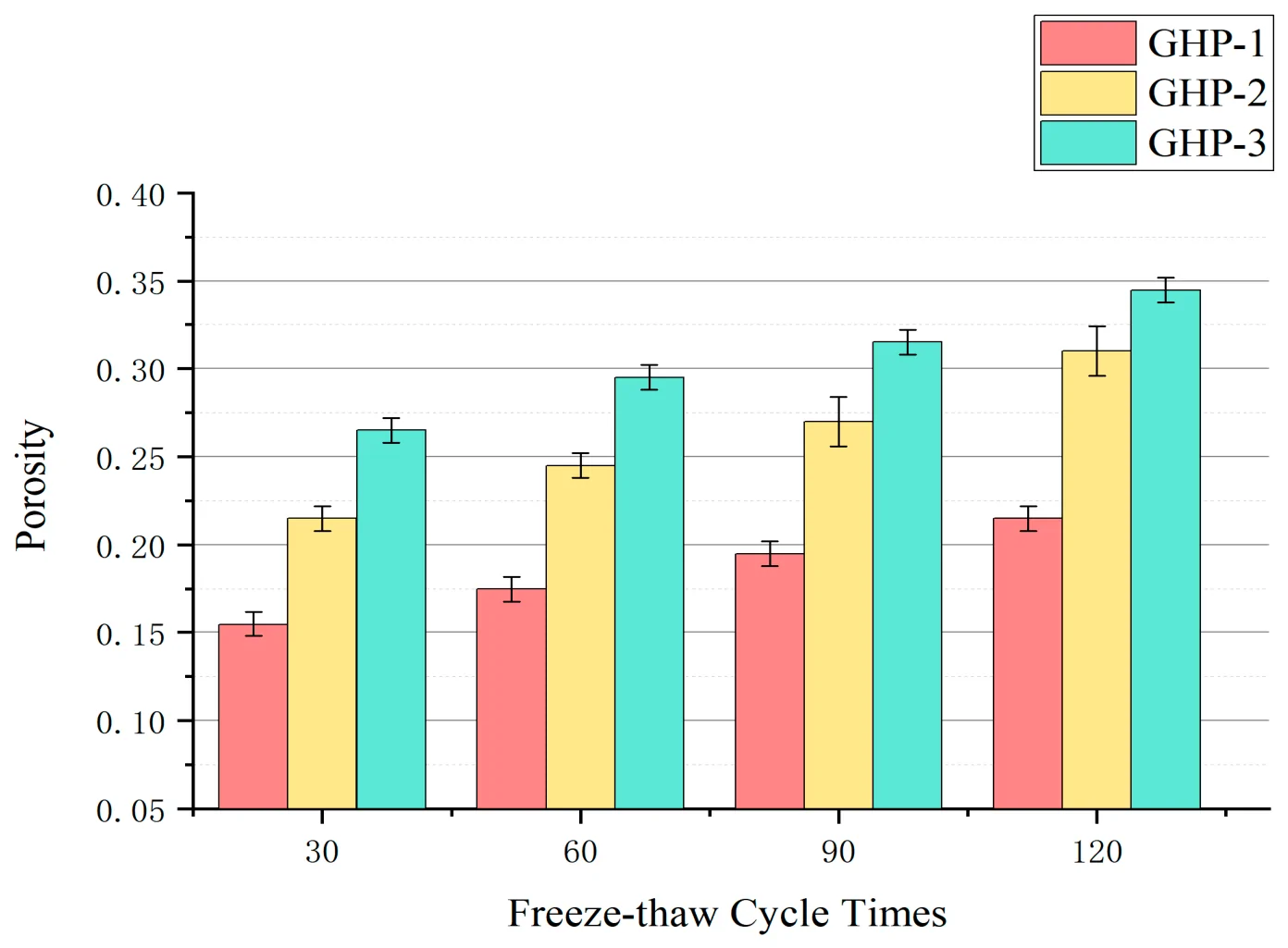
Porosity evolution.

Porosity, as the fundamental structural parameter of permeable concrete, directly determines the quantity, distribution, and connectivity of its internal pores. Consequently, by regulating the phase transition characteristics and internal stress evolution during freeze–thaw cycles, it significantly influences the rate of compressive strength loss and the attenuation rate of the permeability coefficient. As illustrated in Figure 6, the porosity of permeable concrete with varying initial porosities consistently increases with the number of freeze–thaw cycles. After 120 freeze–thaw cycles, the porosity of specimens with initial porosities of 0.15, 0.20, and 0.25 increased to 0.22, 0.31, and 0.35, respectively, representing increases of 42%, 57%, and 40% from the initial values. Furthermore, a higher initial porosity correlates with greater porosity levels in the specimens post-freeze–thaw cycles, leading to more pronounced degradation of the pore structure. This phenomenon can be attributed to specimens with high porosity, which exhibit lower binding of the paste, a smaller effective bonding area, and a relatively loose skeletal structure. Consequently, the freeze expansion stress tends to concentrate locally, thereby facilitating more significant degradation of the pore structure.

Although higher initial porosity reserves abundant drainage channels, it accelerates pore-structure expansion under freeze–thaw cycles. Accordingly, simply increasing porosity cannot guarantee long-term drainage performance; trade-offs between initial pore capacity and frost-resistant stability must be considered for optimal pore-structure determination.

### 3.3. Slip Resistance Performance

**Figure 7:**
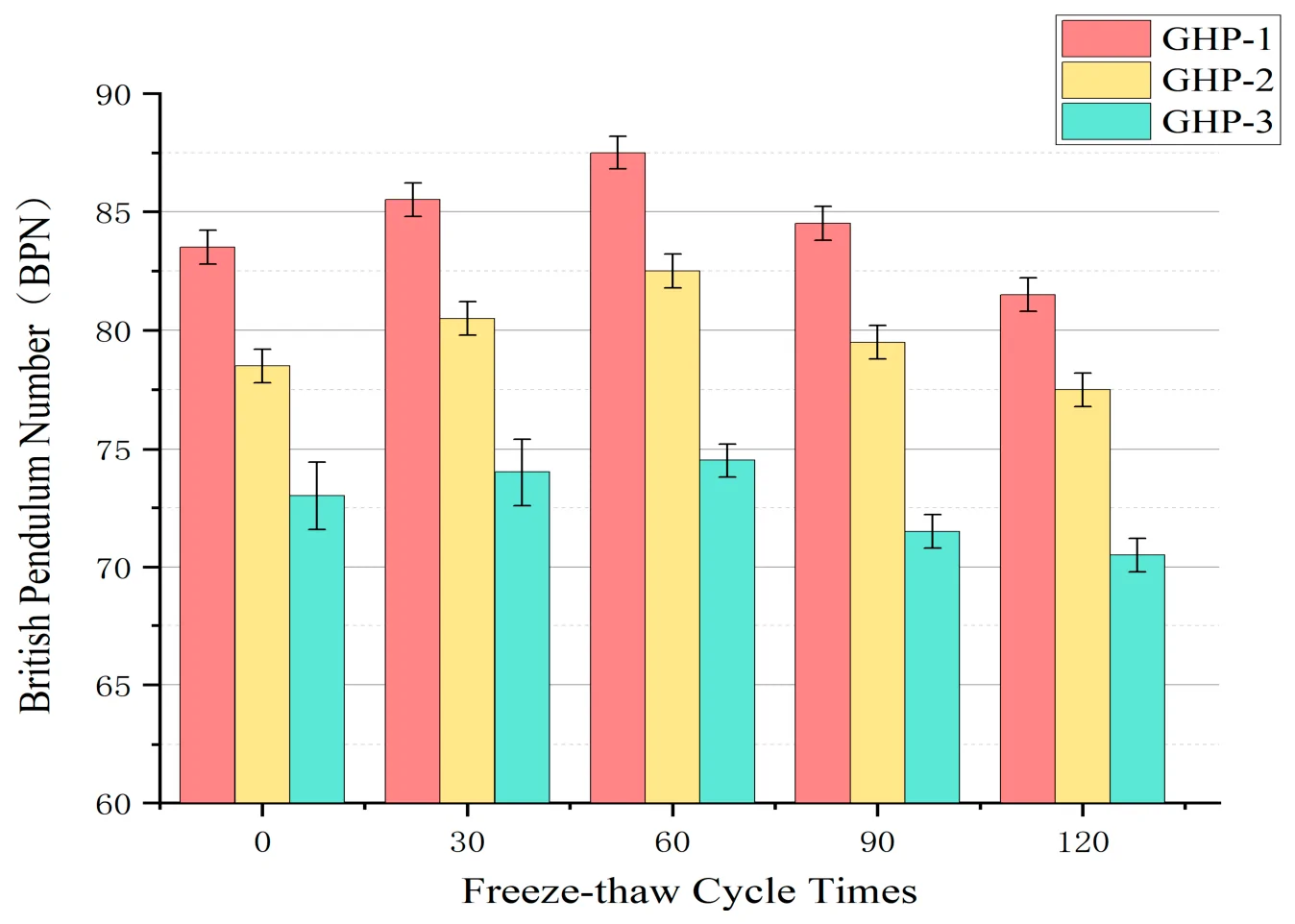
BPN change.

Figure 7 illustrates the variation in BPN of permeable concrete with different porosities under freeze–thaw cycles. Prior to the freeze–thaw cycles, the BPN values for specimens with porosities of 0.15, 0.20, and 0.25 were 83.5, 78.5, and 73.0, respectively. These values decreased progressively with increasing initial porosity, indicating a negative correlation between porosity and initial slip resistance performance. Specimens with lower porosity exhibited a denser distribution of surface aggregates and more pronounced textures, resulting in higher initial friction levels.

With the accumulation of freeze–thaw cycles, all three groups of specimens demonstrated a pattern of BPN values that initially increased and then subsequently decreased. At 60 cycles, BPN values increased to 87.2, 82.3, and 73.5, respectively, indicating slight erosion of the surface paste, resulting in a brief increase in surface roughness. By the time 90 cycles were reached, the surface skeleton began to loosen gradually, and local paste delamination intensified, with BPN values decreasing to 83.4, 79.1, and 72.2, respectively. After 120 freeze–thaw cycles, the BPN values for the three groups of specimens dropped to approximately 81.5, 77.5, and 70.5, with the overall decline slowing down. This indicates that the deterioration of slip resistance performance in permeable concrete has approached saturation due to freeze–thaw cycles. Overall, specimens with an initial porosity of 0.15 exhibited the highest BPN values throughout the freeze–thaw cycles. Although freeze–thaw cycles caused a certain degree of surface structural degradation, after 120 cycles, all groups maintained BPN values greater than 70, which are significantly above the slip resistance requirements of no less than 45 for pedestrian walkways and 55 for vehicular pavements in municipal permeable roadways.

Low-porosity specimens maintain higher BPN values, yet reduced porosity sacrifices part of drainage capacity. This contradictory trend highlights that skid-resistance performance cannot be pursued independently, and it should be coordinated with permeability and frost resistance during pore-structure optimization.

### 3.4. Permeability Performance

**Figure 8:**
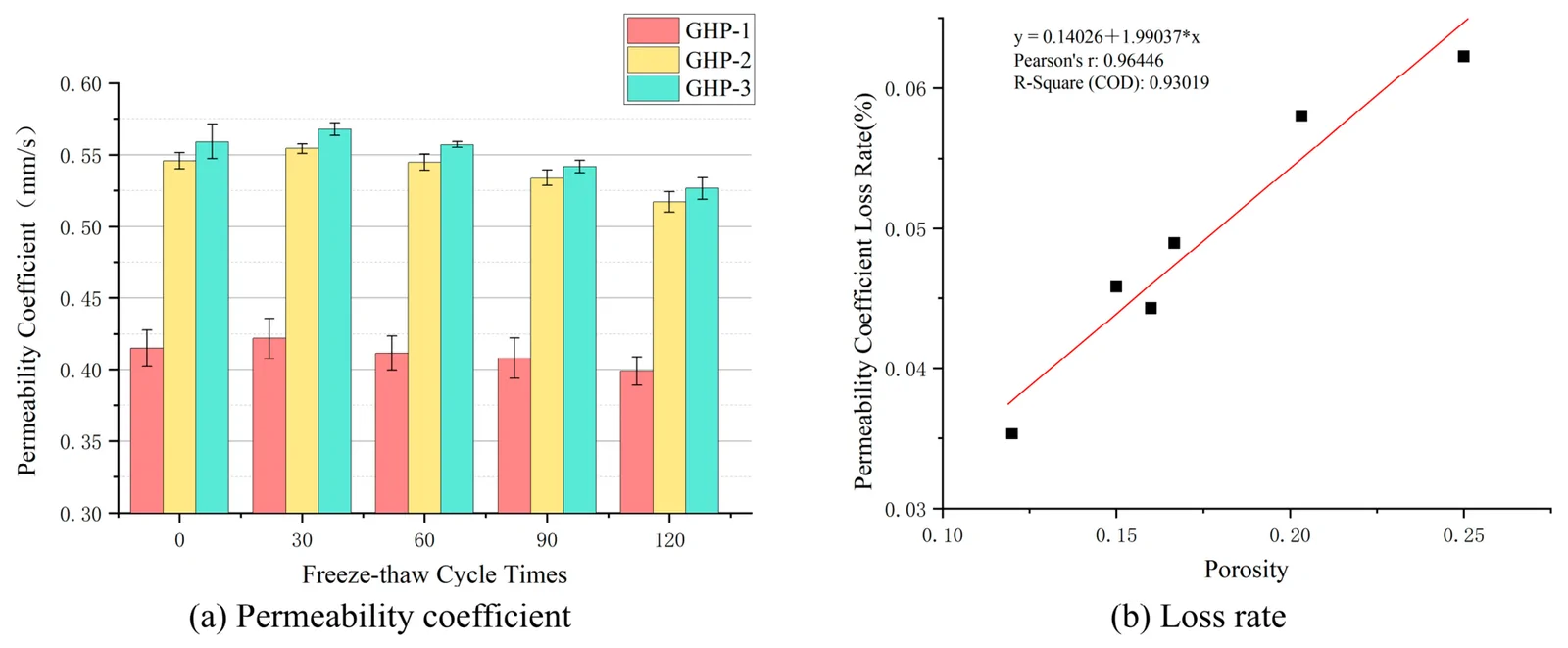
Permeability change.

Figure 8 illustrates that the initial permeability of permeable concrete increases with the rise in porosity. Prior to freeze–thaw cycles, the permeability coefficients of specimens with porosities of 0.15, 0.20, and 0.25 were approximately 0.42, 0.55, and 0.56 mm/s, respectively. This is attributed to the fact that specimens with higher porosity have more interconnected voids and smoother flow pathways, resulting in superior initial permeability.

As the number of freeze–thaw cycles accumulated, the permeability coefficients of all specimen groups exhibited a general declining trend. Upon reaching 120 cycles, the permeability coefficient for the group with 0.15 porosity decreased from 0.42 mm/s to 0.40 mm/s. Meanwhile, the permeability coefficient for the group with 0.25 porosity decreased from 0.56 mm/s to 0.53 mm/s. For specimens with high porosity, the thinner pore walls and weaker framework constraint make them more susceptible to localized damage and the formation of clogging particles under freeze–thaw conditions, leading to a relatively rapid decline in permeability. In contrast, specimens with low porosity possess a denser internal structure and a more stable pore network, resulting in a more gradual decrease in permeability coefficients.

Further analysis indicates that the rate of permeability loss is significantly positively correlated with porosity. This suggests that porosity not only governs the initial level of permeability but also determines the extent of deterioration in permeability after freeze–thaw cycles. In summary, although freeze–thaw cycles weakened permeability, all specimen groups maintained a high level of permeability even after 120 cycles, with no significant failure in drainage functionality.

Elevated porosity improves initial permeability but accelerates permeability attenuation under freeze–thaw action. To obtain balanced service performance, pore-structure design should avoid excessively high porosity even if high initial permeability is required.

### 3.5. Compressive Strength Performance

**Figure 9:**
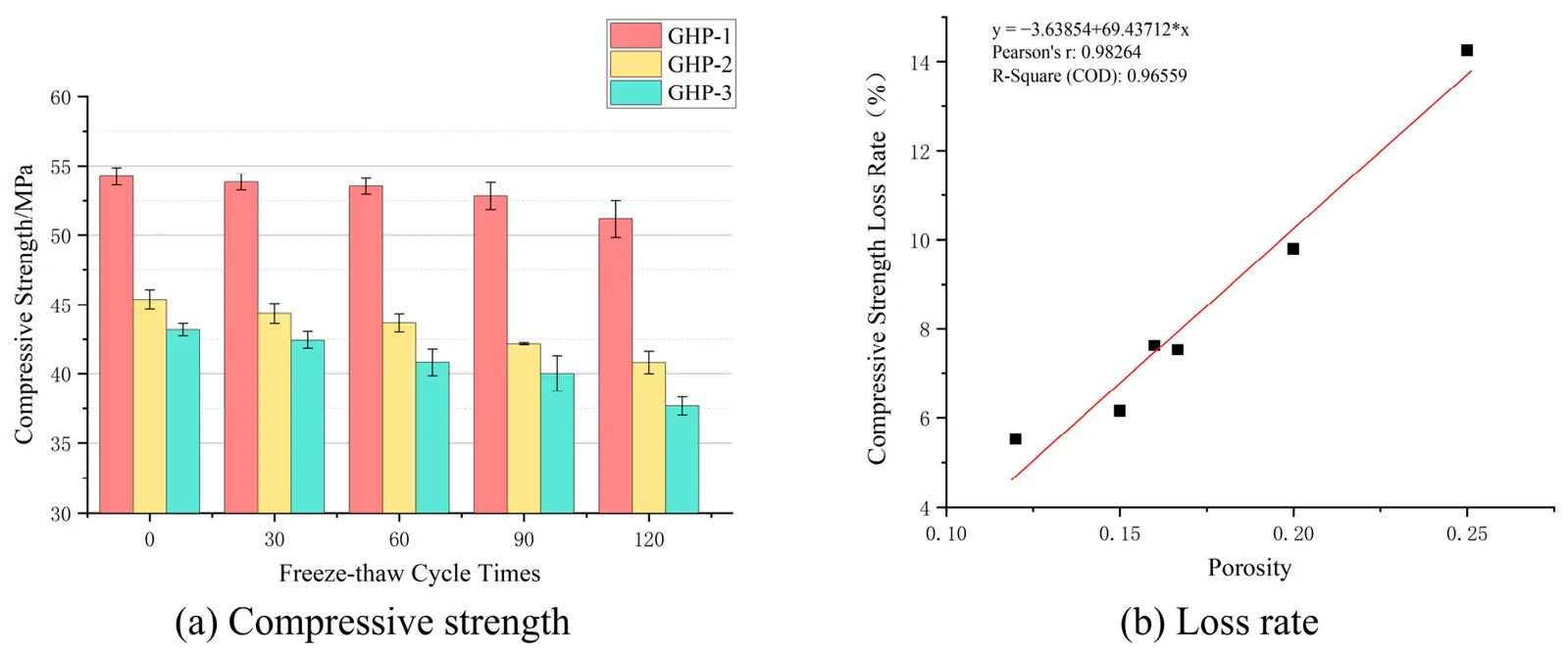
Compressive strength change.

Figure 9 illustrates the variation in compressive strength of permeable concrete with different porosities under freeze–thaw cycles. Before the freeze–thaw cycles, the initial compressive strength of permeable concrete exhibited a significant dependency on porosity, generally decreasing with increased porosity. The initial compressive strengths of specimens with porosities of 0.15, 0.20, and 0.25 were 54.5, 45.6, and 43.3 MPa, respectively, with the 0.15 group showing the highest strength, about 19.5% and 25.9% higher than the 0.20 and 0.25 groups. This indicates that lower porosity corresponds to a denser internal framework and stronger bonding between aggregates and the paste, thus providing the material with a higher initial load-bearing capacity.

With the accumulation of freeze–thaw cycles, the compressive strength of all three groups of specimens consistently decreased, which indicates a significant cumulative degradation effect of freeze–thaw action on the mechanical performance of permeable concrete. After 120 freeze–thaw cycles, the compressive strengths of the 0.15, 0.20, and 0.25 groups decreased to 51.5, 40.9, and 37.7 MPa, respectively, representing reductions of 5.5%, 10.3%, and 12.9% from the initial values. In high porosity specimens, the relatively loose internal framework, thinner pore walls, and smaller effective bonding area lead to easier stress concentration in localized areas, enabling rapid microcrack propagation, resulting in faster strength degradation. In contrast, low porosity specimens have a denser internal structure that can more effectively dissipate freeze–thaw internal stresses, thus maintaining higher structural integrity and mechanical stability.

Further analysis indicates that the rate of compressive strength loss has a significant linear positive correlation with porosity. As porosity increases, the strength loss rate significantly rises, indicating that porosity not only determines the initial strength level but also directly affects the material’s sensitivity to freeze–thaw damage. High porosity specimens exhibit higher mechanical degradation rates because of their loose internal framework and thinner pore walls, which make stress concentration more likely and allow microcracks to propagate and connect more easily during freeze–thaw cycles. This finding further indicates that the pore structure is a key parameter in controlling the freeze–thaw mechanical durability of permeable concrete.

Compressive strength decreases markedly with rising porosity. Mechanical bearing capacity sets a lower limit for pore-structure design; sufficient strength retention after freeze–thaw cycles should be satisfied together with drainage and anti-slip requirements.

### 3.6. Microscopic Damage Mechanism

**Figure 10:**
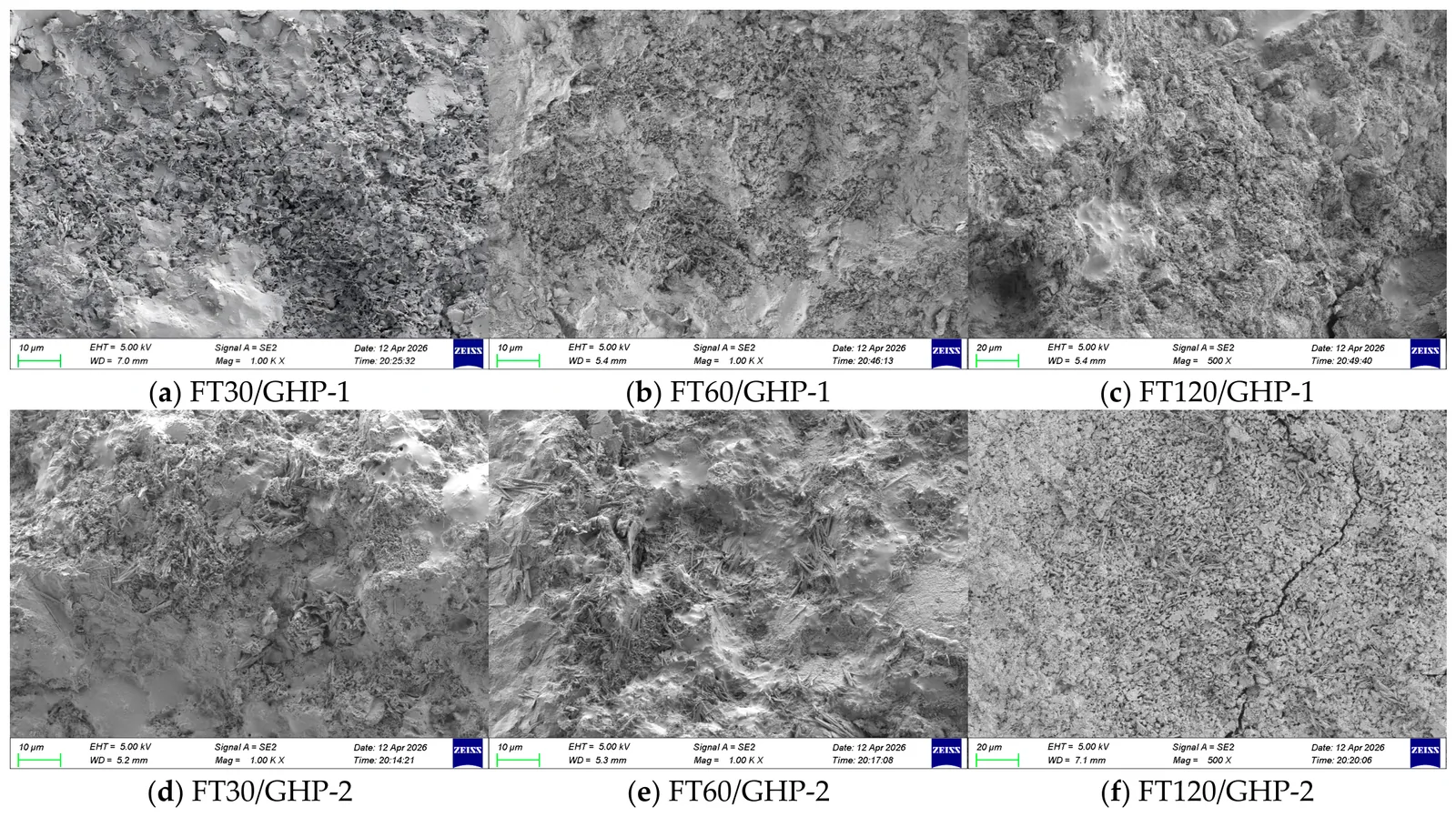

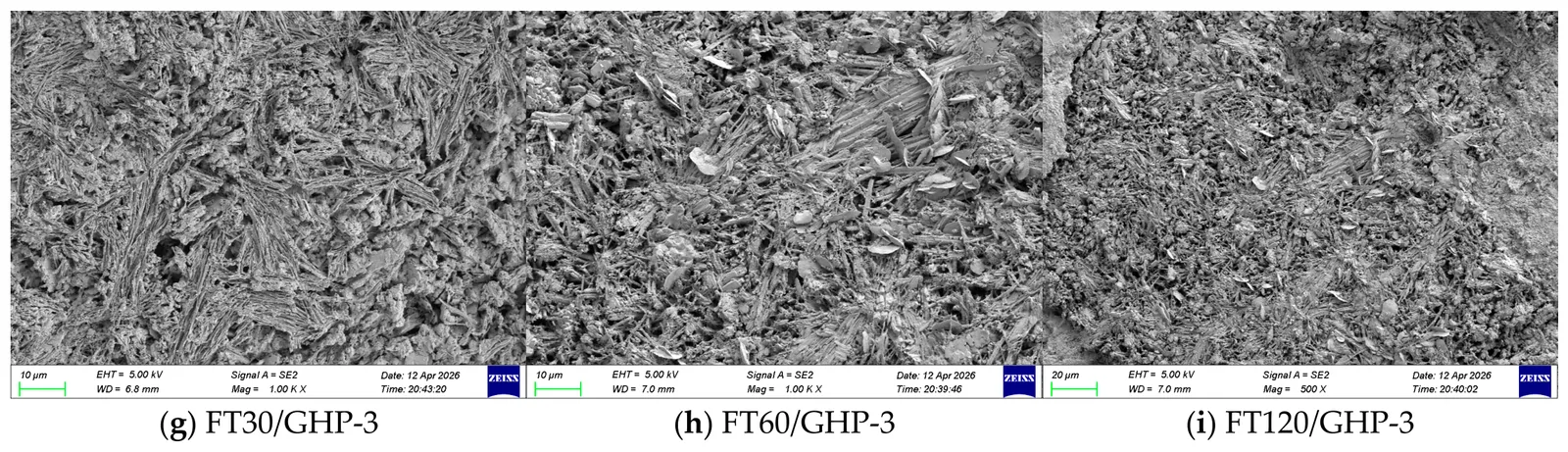
Evolution of the microstructure of permeable concrete.

As the number of freeze–thaw cycles increases, the microstructure of permeable concrete exhibits a gradual degradation characteristic, evolving from localized damage to overall disintegration, as illustrated in Figure 10. During the initial stages of freeze–thaw cycles, the cement matrix remains relatively intact, with needle-like ettringite interwoven and tightly enveloped by C-S-H gel, while minor surface cracks and flaking become apparent. Among the samples, GHP-1 exhibits the highest density, whereas the surfaces of GHP-2 and GHP-3 are comparatively rough. After 60 cycles, the hydrostatic pressure generated by the repeated freezing of pore water, along with osmotic pressure, leads to the degradation of the paste, resulting in a loose and rough surface, localized flaking of the C-S-H gel, and the beginning of exposure and even fracture of the internal crystalline network. By the 120th cycle, the accumulated freeze–thaw stress exacerbates the collapse of the hydration product framework, leading to the accumulation of numerous loosely detached fragments in the matrix, while microcracks continuously propagate to form crack zones. In the case of the GHP-3 specimen, a significant number of isolated, sand-like crystals and gel clusters indicate that the cementitious material has lost its fundamental bonding strength and structural integrity.

The SEM images show localized microcracking and deterioration of the paste–aggregate interfaces after freeze–thaw cycling, particularly in the higher-porosity specimens. These observations are physically consistent with the measured reduction in compressive-strength retention because higher porosity reduces the effective load-bearing area and produces thinner paste bridges.

The micro-scale deterioration phenomena qualitatively interpret the macroscopic experimental trends, i.e., increased porosity, decreased permeability, and reduced compressive strength with increasing freeze–thaw cycles. Porosity directly determines the initial density of the cement paste and its underlying microscopic mechanisms for resisting freeze–thaw stresses. At low porosity levels, the material possesses a denser matrix and a more effective load-bearing cross-section, allowing it to rely on its inherent strength to withstand freeze–thaw damage, thus maintaining a certain degree of integrity even after 120 cycles. Conversely, as porosity increases, the effective encapsulating layer of paste on the aggregate surface significantly thins, leading to a substantial reduction in the load-bearing capacity of the framework. Although highly interconnected pores provide a certain degree of pressure release space for freeze–thaw expansion, overly thin and loose paste layers are unable to withstand cyclical freeze–thaw stresses, making them prone to stress concentration. This, in turn, leads to rapid propagation and penetration of microcracks, ultimately triggering the brittle fracture failure of the bridging structure in the paste.

## 4. Numerical Simulation Analysis

### 4.1. Model Development

**Figure 11:**
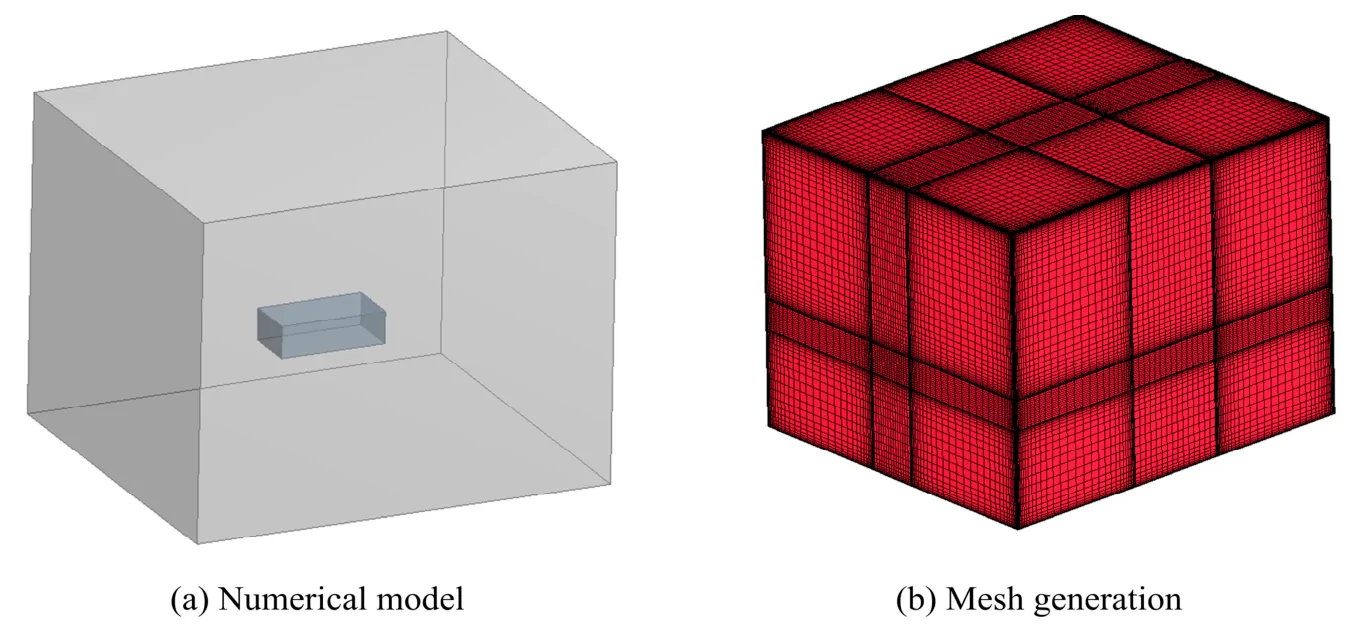
Permeable concrete freeze–thaw model.

This study employs FLUENT software, grounded in computational fluid dynamics theory, to investigate the internal heat transfer and phase change characteristics of permeable concrete with varying porosities during freeze–thaw cycles. The numerical model of the permeable concrete measures 200 mm × 100 mm × 60 mm and is stored in a constant-temperature refrigerator of dimensions 700 mm × 700 mm × 600 mm; the geometric model for the freeze–thaw simulation of the permeable concrete is illustrated in Figure 11a. A structured hexahedral mesh was generated using FLUENT 2021 R2. Local refinement was adopted near the specimen surface and tank wall, with a total of approximately 48,000 cells; Figure 11b shows the computational domain discretized into multiple mesh elements, with local refinement applied near the surface of the permeable concrete and the inner walls of the refrigerator. The thermal and physical parameters of materials involved in the model, including the concrete matrix, water, ice, and air, are critical factors influencing the heat transfer and phase change processes in permeable concrete. The selected key thermal and physical parameters, including thermal conductivity, density, and specific heat capacity of the components used in this study, are presented in Table 5 [41,42,43].

### 4.2. Simulation Methods

In this study, FLUENT 2021 R2 software was employed to investigate the fluid flow and heat transfer characteristics of pore water phase change in permeable concrete during freeze–thaw cycles. The built-in solidification/melting model was selected to simulate the solid–liquid phase transition behavior of internal pore water [44,45,46]. The liquid fraction β was introduced to quantitatively characterize the phase change state of water within porous structures. Specifically, the region with 0 < β < 1 is defined as the mushy zone, which is treated as a porous medium whose local porosity corresponds to the liquid water proportion. Correspondingly, β = 1 represents the pure liquid state of pore water, while β = 0 indicates the complete solid ice state. According to the phase transition properties of pure water, the solidus temperature and liquidus temperature were set to 272 K and 273 K, respectively, forming a narrow 1 K mushy zone to approximate the isothermal phase change behavior of pure water. The enthalpy-porosity

(4) H=h+βL

where h is the sensible enthalpy, β represents the liquid fraction of pore water, and L is the latent heat of water phase change with a fixed value of 334 kJ/kg. Density variation in pore water during freezing and thawing inevitably induces local flow in the phase change region. To eliminate numerical oscillation and describe flow resistance in the mushy zone, a momentum sink correction term is incorporated into the momentum governing equation, as shown in Equation (5):

(5) $$S=\frac{{\left(1-\beta \right)}^{2}}{\beta^{2}+\varepsilon }A_{mush}(v-v_{p})$$

where ***S*** is the momentum sink term for flow correction in the mushy zone; the mushy zone constant $A_{mush}$ is specified as $10^{5}\;kg/(m^{3}\cdot s)$ to ensure sufficient flow damping in the two-phase transition region; the small constant ε=0.001 is introduced to avoid division by zero during numerical iteration; v and $v_{p}$ represent the fluid velocity and porous medium dragging velocity, respectively.

All numerical calculations are performed with a pressure-based transient solver and the SIMPLE algorithm [47]. The second-order upwind scheme is applied for the spatial discretization of momentum and energy equations to improve the accuracy and stability of numerical solutions. The boundary conditions of the numerical model are strictly set according to the actual freeze–thaw cycle test conditions, and the detailed parameter settings are listed in Table 6, which guarantees the authenticity and comparability of the simulation results.

To simplify the numerical solution process while ensuring the rationality and reliability of the calculation results, the following reasonable assumptions are adopted in the numerical model: (1) The permeable concrete skeleton is regarded as a rigid body without volumetric deformation during freeze–thaw cycles, and only the phase change and heat transfer behaviors of pore water and ice in the connected pores are considered. (2) The pore water inside permeable concrete is uniformly distributed, and the influence of uneven water distribution on phase change and heat transfer is ignored. (3) Only heat conduction and convective heat transfer between the concrete surface and cold air are considered in the freeze–thaw process, and the migration and diffusion of pore water are neglected. (4) The thermophysical properties of the concrete skeleton, water/ice, and air remain constant and do not change with temperature throughout the simulation.

### 4.3. Exploratory Numerical Sensitivity Analysis

#### 4.3.1. Temperature Variation Characteristics During Freezing Process

**Figure 12:**
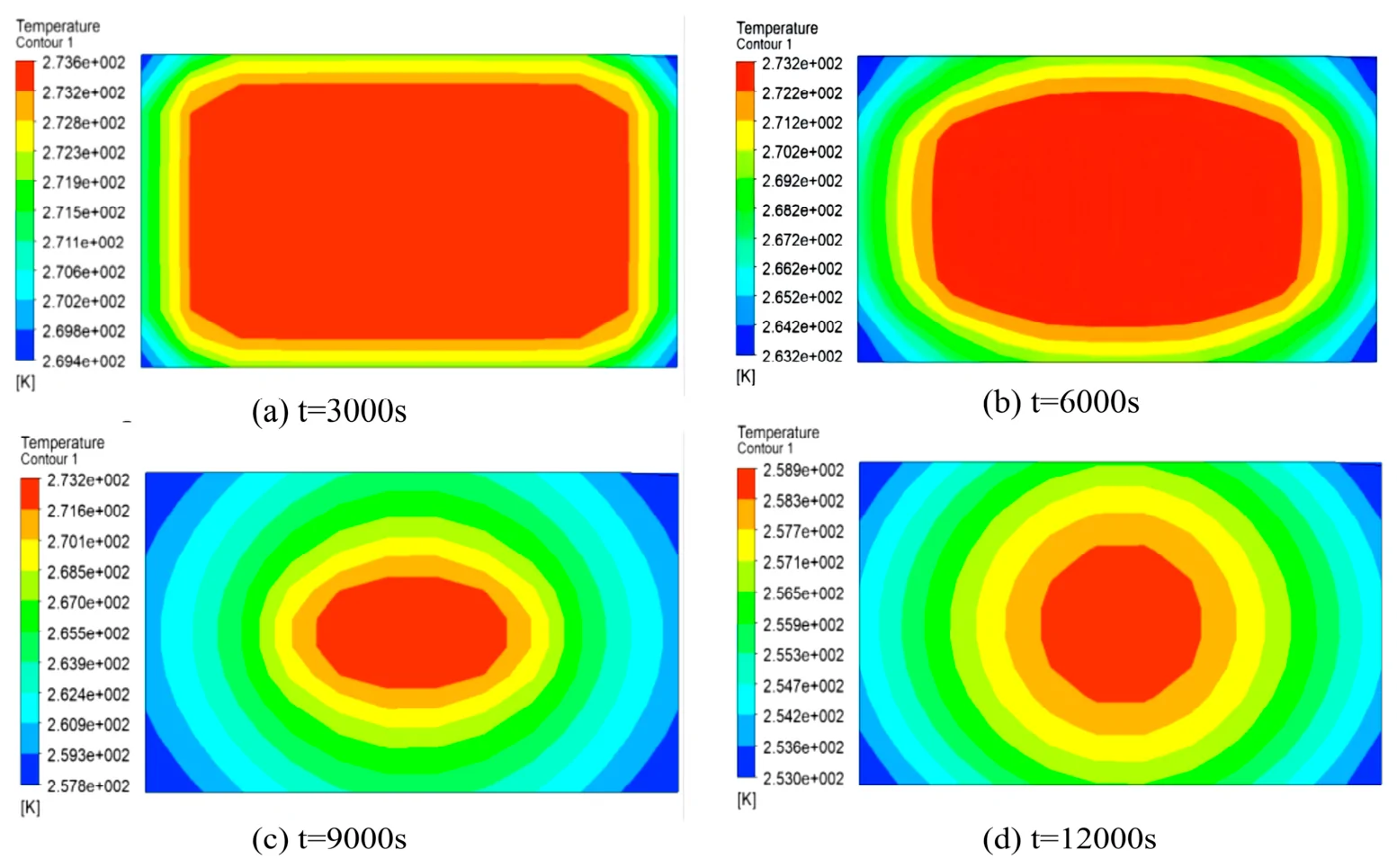
Evolution of the surface temperature of the GPH-1 during freezing.

Figure 12 illustrates the temperature distribution of permeable concrete with a porosity of 0.15 during the freezing process. In the initial cooling phase, the surface temperature first drops to 273 K, forming a circumferential frozen shell. As the cooling duration reaches 9000 s, the outer temperature continues to decrease to 257.8 K due to the extremely cold environment, while the core area maintains a maximum temperature of 273.2 K due to latent heat released from the phase change in pore water. This differential condensation drives the ice front to advance towards the center, causing a rapid contraction of the unfrozen water core. After 12,000 s, when the core’s latent heat is exhausted, the ice front penetrates the axis, transitioning the system into a secondary sensible heat cooling period characterized by temperature difference convergence.

**Figure 13:**
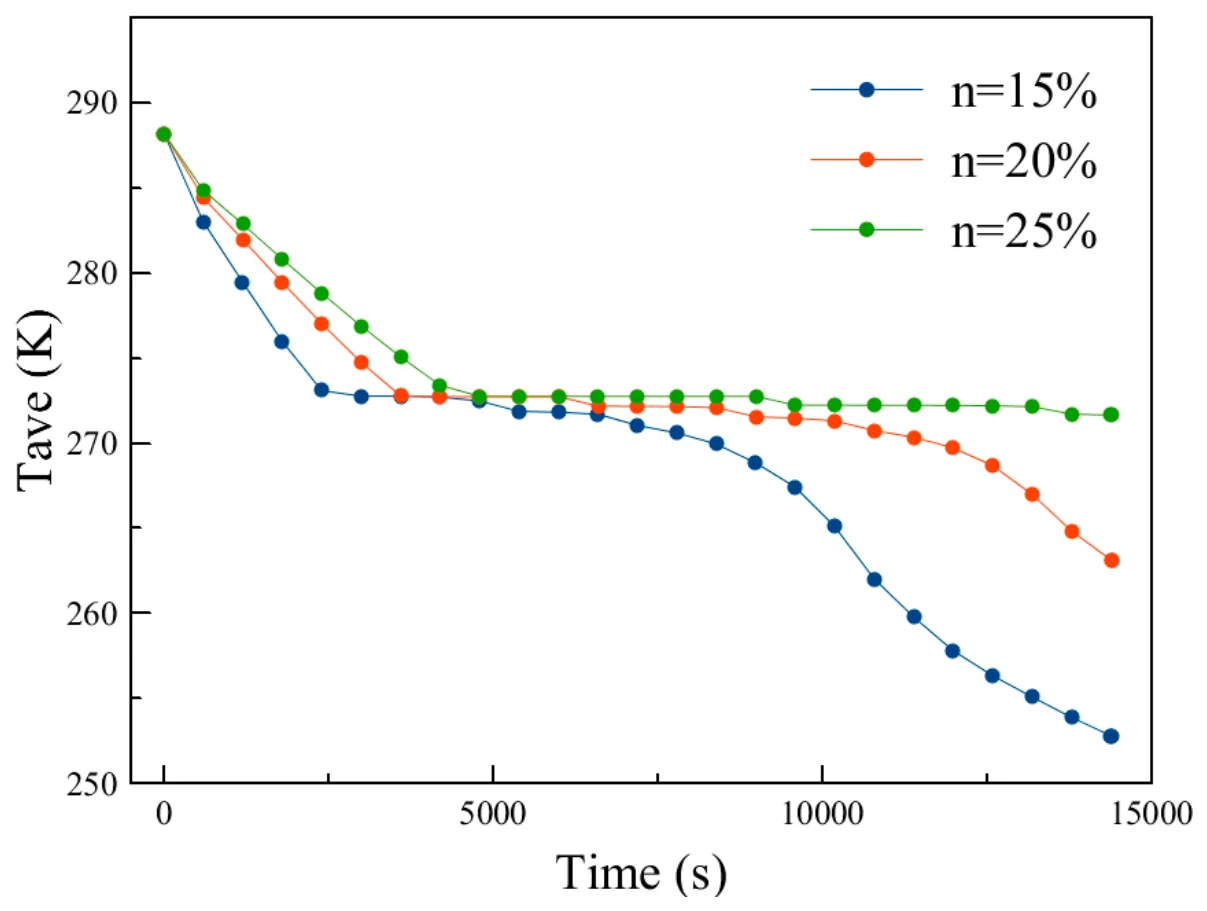
Average temperature evolution.

Figure 13 reveals the temperature response patterns of permeable concrete with varying porosity under freezing conditions, exhibiting a three-stage thermodynamic characteristic primarily governed by phase changes. During the 0–2500 s sensible heat exchange period, driven by the temperature gradient between the material’s interior and the cold environment, sensible heat is dissipated rapidly, resulting in a sharp decline in material temperature; The system then enters a phase change plateau, where the latent heat released from the freezing of pore water effectively compensates for the external cold input, maintaining dynamic thermal balance near 273 K. Once the phase change latent heat is exhausted, the material enters a secondary sensible heat cooling stage, characterized by a rapid decline in temperature.

Further analysis indicates that porosity significantly regulates the heat transfer process. As porosity increases from 15% to 25%, the overall cooling rate is notably delayed, with the phase change plateau extending from 2500 s to 7500 s. This is attributed to the significantly lower thermal conductivity of air compared to that of the concrete framework and the ice-water medium, where increased porosity drastically reduces the effective thermal conductivity of the material, resulting in greatly heightened resistance to heat transfer; Concurrently, high porosity leads to an increased moisture content within the matrix, providing a stronger latent heat reserve for the internal structure. The combined effects of these two factors result in the high-porosity permeable concrete exhibiting a slower cooling rate and a more prolonged phase change hysteresis effect.

**Figure 14:**
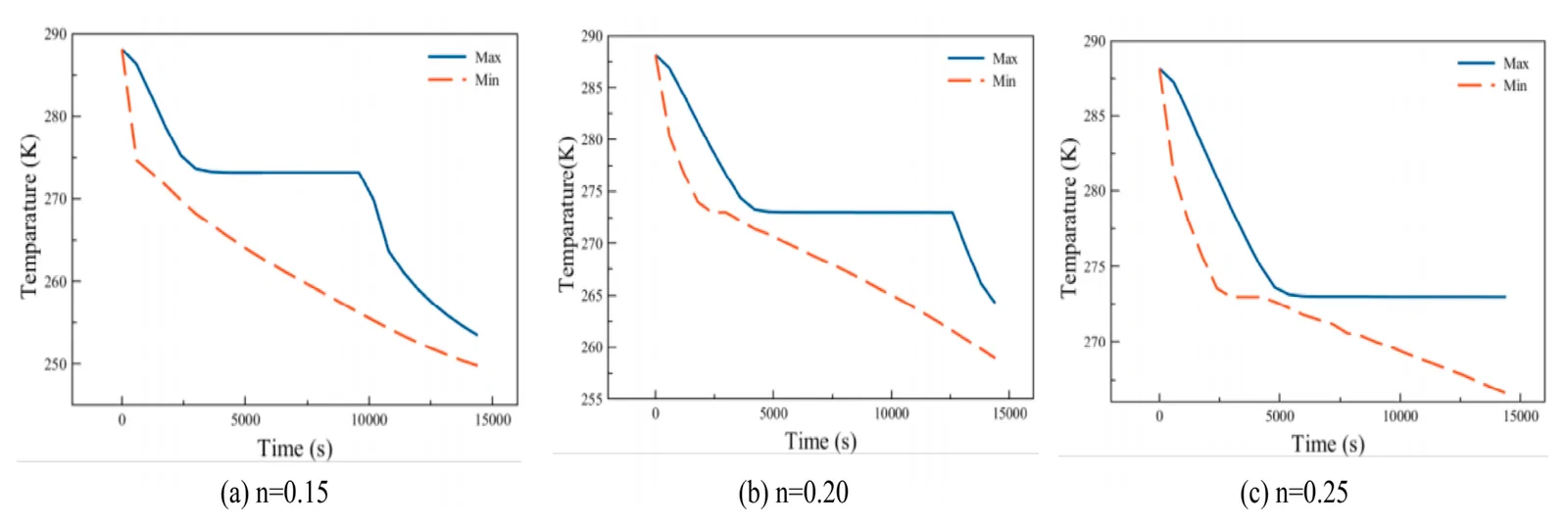
Temperature evolution during the freezing process.

Figure 14 depicts the dynamic evolution of spatial temperature gradients in permeable concrete with different porosities under freezing conditions. During the phase change plateau, the maximum temperature in the core area is maintained at an isothermal level due to latent heat, while the minimum temperature at the surface continues to decline under external environmental influence. This divergence leads to a significant increase in the temperature difference between the interior and exterior. This generates intense spatial temperature gradients within the porous matrix. As porosity increases from 0.15 to 0.25, the termination time of the phase change period extends from approximately 9500 s to around 13,000 s, even spanning the entire observation period of 15,000 s. For high-porosity specimens, this micro-scale thermomechanical coupling damage effect exacerbates cracking in the matrix framework. Essentially revealing why high-porosity specimens exhibit more severe freeze–thaw damage, with significant increases in the reduction rates of compressive strength and permeability.

#### 4.3.2. Temperature Variation Characteristics During Thawing Process

**Figure 15:**
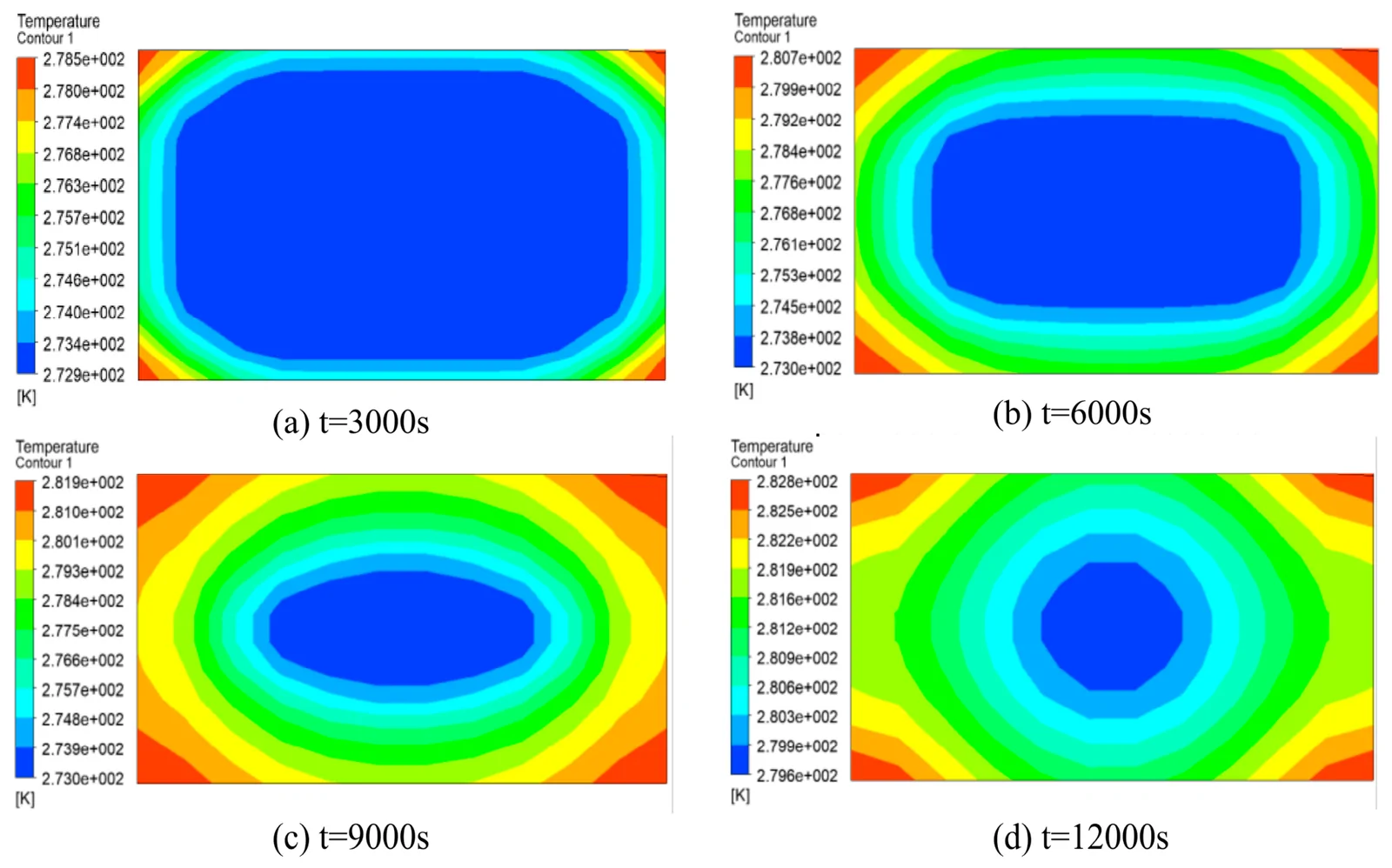
Surface temperature evolution of the GPH-1 during thawing.

As part of the frozen inverse thermodynamic evolution, the thawing process of permeable concrete with a porosity of 0.15 also follows the heat transfer mechanisms of sensible heat heating, phase change heat absorption, and secondary sensible heat heating, with the characteristics of its temperature field evolution illustrated in Figure 15. In the initial thawing phase, driven by external heat flow, the temperature at the specimen’s boundaries and corners first reaches 273 K, forming a closed high-temperature ring, while the core region remains in the sensible heat heating phase below 273 K in the ice. As the heat transfer progresses, the high-temperature boundary continues to expand inward; however, the significant latent heat absorption during the ice-water phase change strongly buffers the heat flow, causing the core temperature to remain at around 273 K during the phase change plateau. This results in the generation of a significant temperature gradient within the specimen. At this stage, the macroscopic solid–liquid coexisting state formed by the outer liquid water and the residual ice in the core easily induces and exacerbates the complex coupling effects between thermal stress and phase change contraction stress. When the thawing evolves to 12,000 s, the melting front converges and completely penetrates the center of the specimen, with the internal temperature difference beginning to converge after reaching its peak. At this point, the phase change process comes to a complete end, and the system as a whole transitions into the secondary sensible heat heating range, where liquid water and the concrete skeleton work in synergy, progressing the thermodynamic state of the specimen toward a gradual equilibrium with the environmental temperature of 290 K.

**Figure 16:**
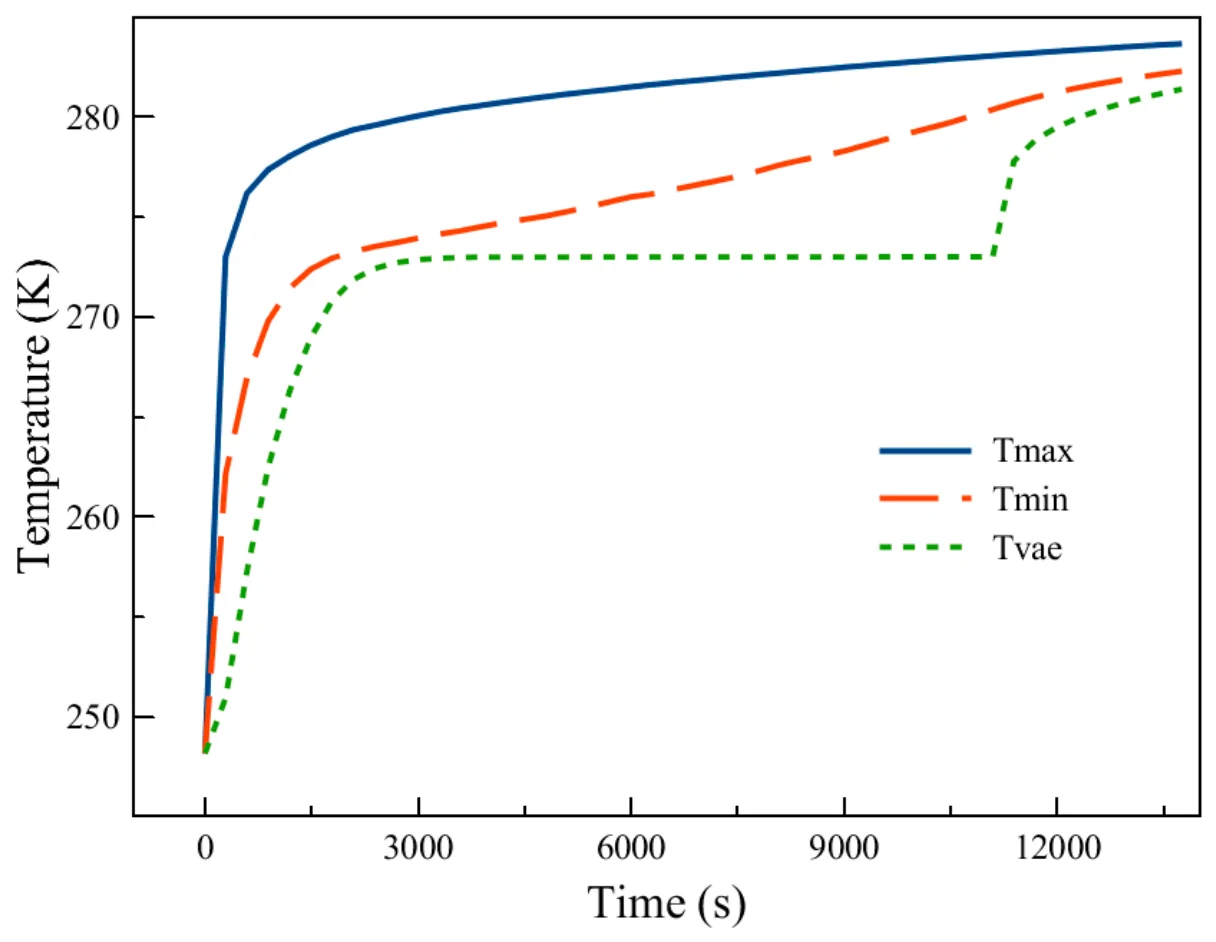
Thaw-period temperature fluctuations.

Figure 16 illustrates the evolution of the maximum temperature (Tmax), minimum temperature (Tmin), and average temperature (Tave) during the thawing of permeable concrete with a porosity of 0.15. In the initial phase, driven by a strong temperature gradient, the system experiences a rapid increase in sensible heat. Subsequently, during the thawing interval from 2500 to 11,000 s, the melting of pore ice absorbs a substantial amount of latent heat, resulting in a significant thermal buffering effect that leads to the formation of a phase change plateau in the Tave curve around 8500 s. During this period, the asynchronous melting mechanism from the surface to the interior leads to an intensified differentiation between Tmax and Tmin, resulting in a peak internal temperature difference that induces strong non-uniform thermal stresses. When t exceeds 11,000 s, the ice completely melts, and the latent heat of phase change is depleted. The system transitions into a secondary sensible heat heating phase characterized by liquid–solid cooperation, where the internal and external temperature differences rapidly converge toward an equilibrium state with the environment. Overall, the evolution of Tave maps the energy conversion trajectory during the solid–liquid phase change of porous media, providing crucial insights for assessing transient heat transfer and thermal stability within complex pore networks.

### 4.4. Limitations

Several limitations of the present numerical analysis should be acknowledged. First, the model lacks experimental temperature validation; the simulated temperature histories, phase-change durations, and internal temperature gradients have not been compared with measured data. Second, the model assumes a rigid skeleton, constant thermophysical properties, uniform initial moisture distribution, and pure-water phase change at a fixed temperature, which may deviate from the real freeze–thaw process where moisture migration, supercooling, temperature-dependent properties, and dissolved salts affect the phase-change behavior. Third, the model does not solve the mechanical field, and therefore cannot directly predict thermomechanical damage. The numerical results should therefore be regarded as qualitative trends obtained under idealized conditions, rather than quantitative predictions. Future work should incorporate embedded temperature measurements, moisture transport, temperature-dependent properties, and mechanical coupling to improve the model’s predictive capability.

## 5. Conclusions

This paper focuses on permeable concrete with varying porosities, conducting 120 low-temperature freeze–thaw cycle tests to systematically evaluate its anti-slip performance, permeability, compressive strength, and the evolution of its pore structure. Additionally, employing CFD numerical simulation methods, the study reveals the internal temperature field distribution of permeable concrete during freeze–thaw and thawing processes, as well as the characteristics of the evolution of freezing and melting fronts, clarifying the intrinsic mechanisms of how porosity affects freeze–thaw damage.

The primary conclusions are as follows:

(1) The failure of permeable concrete due to freeze–thaw cycles essentially involves a progressive process, ranging from localized peeling of the C-S-H gel and the initiation of microcracks to the complete disintegration of the crystalline network. High porosity specimens are particularly susceptible to failure because the thin layer of paste effectively encasing the aggregate surfaces produces weak internal structural constraints. Under the repeated frost heave stresses induced by the ice-water phase change, stress concentrations are readily initiated, ultimately resulting in the rupture of the cement paste bridging structure.
(2) After undergoing 120 freeze–thaw cycles, the mass loss of all specimens remained below 1%; however, the compressive strength decreased by 5.5% to 12.9%. For specimens with higher porosities, internal pore structure deterioration was more pronounced, with the rates of loss in compressive strength and permeability positively correlated with the initial porosity.
(3) The BPN values on the surfaces of the specimens exhibited an initial increase followed by a decrease during the freeze–thaw cycle period. Although the local particle detachment due to freeze–thaw cycles caused a slight reduction in permeability, all groups of specimens maintained BPN values above 70 after 120 cycles, indicating no substantial failure in their drainage functionality.
(4) Exploratory numerical simulations, conducted under the idealized assumptions stated in Section 4.2, suggest that the thermal response of permeable concrete during freeze–thaw cycles is porosity-dependent. Under the current idealized conditions, the simulated phase-change plateau extended from approximately 2500 s to 7500 s as porosity increased from 15% to 25%. These trends are consistent with the observed frost damage patterns but should be interpreted as qualitative indications rather than quantitative predictions.

## Figures and Tables

**Table 1:** Chemical composition of cementitious materials.

Type	Chemical Composition (%)									
	CaO	SiO_2_	Al_2_O_3_	Fe_2_O_3_	SO_3_	MgO	K_2_O	Na_2_O	TiO_3_	Loss
Cement	62.51	21.62	5.21	3.52	2.82	1.83	0.81	0.32	0.30	1.07
Fly ash	3.81	62.21	28.12	0.75	0.89	1.24	0.59	0.26	0.98	2.15

**Table 2:** Physical parameters of coarse aggregate.

Apparent Density (kg/m^3^)	Bulk Density (kg/m^3^)	Particle Size (mm)	Silt Content (%)	Needle-Like Aggregate Mass Fraction (%)
2755	1540	5~10	0.35	7.5

**Table 3:** Physical parameters of river sand.

Fineness Modulus	Apparent Density (kg/m^3^)	Bulk Density (kg/m^3^)	Silt Content (%)
2.2	2673	5~10	1.9

**Table 4:** Mix design.

Group	Target Porosity (%)	Measured Porosity (%)	Cement (kg/m^3^)	Fly Ash (kg/m^3^)	Gravel (kg/m^3^)	Sand (kg/m^3^)	Water (kg/m^3^)	Water Reducer (kg/m^3^)
GHP-1	0.15	0.139	447.88	111.97	974.15	705.42	190.35	1.68
GHP-2	0.20	0.212	335.91	83.98	974.15	705.42	142.46	1.26
GHP-3	0.25	0.264	223.94	55.98	974.15	705.42	102.18	0.97

**Table 5:** Material parameters.

	Viscosity Pa·s	Specific Heat J/(kg·K)	Density kg/m^3^	Thermal Conductivity W/(m·K)
Air	1.8 × 10^−5^	1005	1.2	0.026
Water	1.0 × 10^−3^	4182	998	0.6
Ice (solid)	—(solid)	2090	917	2.2
Concrete skeleton	—(solid)	850	1900	1.2

**Table 6:** Boundary and initial conditions.

Parameter	Freezing Stage	Thawing Stage
Specimen initial temperature	15 °C (288 K)	—(end state of freezing)
Chamber air temperature	−25 ± 2 °C (248 K)	+5 ± 2 °C (278 K)
Chamber wall temperature	−30 °C (243 K)	+5 °C (278 K)
Convective heat transfer coefficient	15 W/(m^2^·K)	15 W/(m^2^·K)
Duration	4 h (14,400 s)	2 h (7200 s)
Water–ice phase-change temperature	0 °C (273 K)	0 °C (273 K)
Solver	Pressure-based, transient, SIMPLE	
Specimen initial temperature	15 °C (288 K)	—(end state of freezing)
Time step	1.0 s	0.5 s
Convergence criteria	Continuity: 10^−3^; Momentum: 10^−3^; Energy: 10^−6^	
Mesh (medium)	~48,000 hexahedral cells (ICEM CFD)	

## Data Availability

The original contributions presented in this study are included in the article. Further inquiries can be directed to the corresponding authors.
